# Human gut microbiota: the links with dementia development

**DOI:** 10.1007/s13238-016-0338-6

**Published:** 2016-11-19

**Authors:** Rashad Alkasir, Jing Li, Xudong Li, Miao Jin, Baoli Zhu

**Affiliations:** 1 Institute of Microbiology, Chinese Academy of Sciences, Beijing, 100101 China; 2 China-Japan Friendship Hospital, Beijing, 100029 China; 3 Collaborative Innovation Centre for Diagnosis and Treatment of Infectious Diseases, The First Attainted Hospital College of Medicine, Zhejiang University, Hangzhou, 310058 China

**Keywords:** dementia, alzheimer’s disease, gut microbiota, inflammation, probiotics

## Abstract

Dementia is a comprehensive category of brain diseases that is great enough to affect a person’s daily functioning. The most common type of dementia is Alzheimer’s disease, which makes most of cases. New researches indicate that gastrointestinal tract microbiota are directly linked to dementia pathogenesis through triggering metabolic diseases and low-grade inflammation progress. A novel strategy is proposed for the management of these disorders and as an adjuvant for psychiatric treatment of dementia and other related diseases through modulation of the microbiota (e.g. with the use of probiotics).

## Introduction

Dementia is a syndrome that affects memory and other cognitive functions to the extent that it interferes with daily function. There are many conditions that can cause dementia, including neurodegenerative disorders [e.g. Alzheimer’s disease (AD) and Parkinson’s disease (PD)], cerebrovascular disease, brain injury, and certain infections. AD is the most prevalent neurodegenerative disorder and the leading cause of dementia worldwide, accounting for approximately 60%–70% of all cases (Irizarry, 2001; Fratiglioni, 2001). AD is a highly incapacitating disorder, progressing from minor memory problems to a complete loss of mental functions and in the long run, resulting in death. The symptoms of AD are caused by a progressive loss of cholinergic function due to neuronal cell death in the hippocampus cerebral cortex and different regions of the brain which regulate thought process and memory. Furthermore, the neuropathological hallmarks of AD consist of two kinds of protein aggregates, amyloid beta (Aβ) plaques and hyper-phosphorylated tangles of tau-protein. Aβ is a transmembrane protein which has no known function, which is constitutively cleaved into peptides during the metabolic functions of the cell (Haass et al., 1992). However, in the case of overproduction (or impaired clearance), Aβ aggregates into extracellular oligomers, fibrils and eventually, plaques (Masters et al., 1985). Whereas, *tau* is an intracellular microtubule binding protein which, when hyper-phosphorylated, results in the disassembly of microtubules and thus leads to the impediment axonal transport and compromises neuronal and synaptic functions (Iqbal et al., 2005). There are now several studies linking obesity and other metabolic disorders with an increased risk of AD. In addition, the inflammation and the pathogens interaction hypothesis (i.e. microbial infections are causing the tau tangles and build-up of amyloid proteins, resulting in the ensuing cell death) were also risk factors of AD development.

The ‘gut microbiota’ can be defined as all the species within the ecosystem and are considered the largest reservoir of microbes in the human body, containing about 10^14^ microbes. Over 99% of microbiota in the GI tract are anaerobic bacteria, with archaebacterial, protozoa, fungi, and other microorganisms making up the remainder (Hill et al., 2014). These GI microbiota play many important roles in physiological homeostasis and metabolism, conferring many health benefits on the host organism, including pathogen displacement, immune system development, barrier fortification, vitamin production and nutrient absorption, and is often referred to as the ‛forgotten organ’ as a result of what it is (O’Hara and Shanahan, 2006). Consequently, there is a great deal of impetus for the comprehensive understanding of the complete pathological function, genetic information and functional diversity of the gut microbiome that may lead to clinical AD and the discovery of variable risk factors (Clemente et al., 2012).

In this review, we examine the roles of the gut microbiota in the maintenance of health, inflammation process; and how it is implicated in acquired metabolic diseases and aging-related morbidity; highlighting the dementia, particularly in AD regulation. Finally, we discuss the potential modification to providing clinical benefit of the gut microbiome and other methods for AD avoidance.

## Brain-Gut axis

Bidirectional communications between the gut tract and the central nervous system (CNS)—the braingut axis—occur in both sickness and health. Recently, a role of the enteric microbiota which count as both commensal and pathogenic organisms (such as *Bifidobacterium* and *Escherichia*), in the brain–gut axis interactions was essentially identified (Grenham et al., 2011). The gut microbiota assists a number of everyday functions in the brain, including the regulation of the hypothalamic-pituitary-adrenal (HPA) axis activation state. The release of cortisol as a result of HPA axis activation can in turn govern the activation state of brain microglia, and effect cytokine release as well as attracting of monocytes from the periphery to the brain. They also can rule actions in the periphery and central nervous system by various means of communication including vagal nerve and adrenergic nerve activation as well as producing several molecular candidates such as neurotransmitters, neuropeptides, endocrine hormones and immunomodulators. Host stress hormones such as noradrenaline, which might affect bacterial activities or signalling between bacteria, may change the microbial diversity and actions of the gut microbiota. However, these bacteria are capable of synthesizing and releasing many neurotransmitters and neuromodulators themselves, or evoke the synthesis and release of neuropeptides from enteroendocrine cells. For example: *Lactobacillus* and *Bifidobacterium* species can produce short-chain fatty acids; *Escherichia*, *Bacillus* and *Saccharomyces* spp. can produce norepinephrine; spore-forming microbes can produce 5-HT; *Bacillus* can produce dopamine, and *Lactobacillus* can produce acetylcholine (Table 1) (Grenham et al., 2011; Wall et al., 2014; Potgieter et al., 2015; Yano et al., 2015).

**Table 1:** The gut bacteria and their metabolites on the nervous system

**Gut microbiota**	**Metabolites product**	**Effects on the nervous system function**	**References**
*Lactobacillus, Bifidobacterium*	GABA	Inhibitory neurotransmitter, metabolic disorders can lead to anxiety and depression	(Barrett et al., 2012)
*Streptococcus, Escherichia, enterococci, Enterococcus, Lactococcus, Lactobacillus*	Serotonin	Neurotransmitters, regulate emotions	(Shishov et al., 2009; Özogul, 2011)
*Bacillus*	Norepinephrine	Neurotransmitters involved in motor, cognitive, memory, emotion and other central nervous and endocrine control	(Tsavkelova et al., 2000; Shishov et al., 2009)
*Lactobacillus, Bacillus*	Acetylcholine	Acting on neurotransmitters in the central and peripheral nervous systems, and cognitive function, particularly closely related to learning and memory	(Marquardt and Spitznagel 1959;Kawashima et al., 2007)
*Lactobacillus, Lactococcus, Streptococcus, Enterococcus*	Histamine	Regulating neurotransmitter; sleep and cognitive function related	(Landete et al. 2008; Thomas et al., 2012)
*Clostridium, C. sporogenes*	Indole-3-propionic acid (IPA)	Antioxidants, protect neurons	(Jellet et al., 1980;Bendheim et al., 2002)
*Bacteroides, Bifidobacterium, Propionibacterium, Eubacterium, Lactobacillus, Clostridium, Roseburia, Prevotella*	Short-chain fatty acids (SCFA)	Carbohydrates (starch, cellulose, etc.), the main products of fermentation, to provide energy for the host, regulate endothelial cell function, promote the synthesis and secretion of neurotransmitters and hormones, reduce inflammation	(Russell et al., 2013)
Blue-green algae (*Cyanobacteria*)	BMAA	Neurotoxicity, neuronal damage, and misfolded proteins related	(Bradley and Mash, 2009)
Gram-negative bacteria	Endotoxin	Induced inflammation, release large amounts of inflammatory cytokines (TNF-α, IL-6 and IL-8, etc.), obesity, IR, diabetes and is closely related to the occurrence of AD	(Levi et al., 2003;Wang and Quinn, 2010)
*Escherichia, Bacillus, Lactococcus, Lactobacillus, Streptococcus*	Dopamine	System activity, Parkinson’s disease, AD, and depression-related	(Tsavkelova et al., 2000; Shishov et al., 2009; Özogul, 2011)
Spore-forming microbes, *Candida, Streptococcus,* *Enterococcus* spp.	Promote 5-HT biosynthesis	Increase the motility of the gut	(Yano et al., 2015)

NOTE: GABA: gamma-aminobutyric acid; BMAA: beta-N- methylamino -L- alanine; 5-HT: 5-hydroxytryptamine; AD: Alzheimer’s disease; IR: insulin resistance

Dysfunction in the brain–gut microbiota axis was investigated in irritable bowel syndrome, inflammatory bowel disease, depression, and anxiety, as well as neurodevelopmental disorders such as autism, Parkinson’s disease (PD), and AD (Bonaz and Bernstein, 2013; Dinan and Cryan, 2013; Hsiao et al., 2013; Borre et al., 2014b). For example, the recent experimental study of Holmqvist *et al* demonstrated that α-synuclein, the presynaptic neuronal protein which is abundantly expressed in the brain, was presented in PD brain lysate and distinct recombinant α-synuclein forms were transported via the vagal nerve and reached the dorsal motor nucleus of the vagus in the brainstem in a time-dependent manner after injection into the intestinal wall. The authors indicated that different α-synuclein forms can spread from the gut to the brain, and that microtubule-associated transport is joined in the translocation of aggregated α-synuclein in neurons (Holmqvist et al., 2014). In addition, Scheperjans *et al* studied the microbiota composition in PD patients and healthy controls. The results suggested a significant decrease of *Prevotellaceae* in PD patients and the subjects with a lower abundance of *Prevotellaceae* (<6.5% in relative abundance) showed a higher risk of PD. In another study, the abundance of *Enterobacteriaceae* was positively correlated with the severity of PD regarding gait difficulty and postural instability (Scheperjans et al., 2015).

## The role of gut microbiota in ad via the inflammation regulatION

Inflammation is the body’s response to infections and tissue injury and the inflammatory response is orchestrated by the cells of the immune system; both from the “adaptive” branch (including T- and B-cells with the capacity to induce long-term memory of encountered pathogens, “immunisation”) and the “innate” branch (including monocytes, macrophages, dendritic cells, and mast cells, that are targeted against common pathogen antigens). Inflammation was first implicated in AD pathology and development in the 1990s, with the neuropathological finding of activated inflammatory cells (microglia and astrocytes) and inflammatory proteins (e.g. cytokines and complement), surrounding the amyloid plaques and the neurofibrillary tangles (Aisen and Davis, 1994). In addition to the epidemiological findings, patients suffering from arthritis and other patient groups with a high intake of non-steroidal anti-inflammatory drugs (NSAID) were observed to have had a lower proportion of individuals affected with AD. Many of the earliest results were at first dismissed as inaccurate given the perception of the brain as an “immune privileged” organ, i.e. an organ that does not elicit inflammation in response to antigens or damage. However, abundant literature can now be found in relation to the presence of acute phase proteins in Aβ plaques, activated microglial cells that stain for inflammatory cytokines, and components of the complement system in brain tissue of AD patients. Identifying inflammation-associated risk factors for AD could provide clues to the aetiology of AD and lead to novel strategies for combating the disease (Akiyama et al., 2000; Cacquevel et al., 2004). Since the initial discovery of a potential inflammatory ingredient to the AD cocktail, studies have diversified to look at a multitude of inflammation-associated risk factors for cognitive function, cognitive decline, AD, dementia and progression in dementia; including circulating inflammatory markers (Engelborghs et al., 1999; Yaffe et al., 2003; Tan et al., 2007; Zuliani et al., 2007), genetic sequence variation in immune-related genes (Arosio et al., 2004; Flex et al., 2004), and proxies of inflammatory load (Gatz et al., 2006).

Microbiota affect the development of the gut associated lymphoid system (GALT). The intestines contains 70% of the body’s circulating lymphocytes, many of which are found within the epithelium (Collins et al., 2012). In the lamina propria there are several lines of immune cells, key to the host response to microbiota, such as macrophages, dendritic cells and myofibroblasts (Otte et al., 2003; Bilsborough and Viney, 2004). Gut lymphoid tissue, and surface and circulating immunoglobulin concentrations show a substantial rise in observation of bacterial addition to the gut (Macpherson and Harris, 2004). In the early stages of the human life cycle, pioneering species in the gut interact through surface cell receptors on the immune cells of the gut, such as caspase-recruitment-domain protein (CARD), and toll-like receptors (TLRs), to promote the expression of host genes that generate an intraluminal and mucosal environment that further favors their colonization (Silva et al., 1987; Hooper et al., 2001). In addition to the TLRs there is another family of membrane-bound receptors for detection of proteins called NOD-like receptors (NLRs). NRLs are located in the cytoplasm and are involved in the detection of bacterial pathogen-associated molecular patterns (PAMPs) that enter the mammalian cell. NRLs are especially important in tissues where TLRs are expressed at low levels (Philpott et al., 2001). In addition to intestinal epithelial cells, the epithelium includes specialized cells such as goblet cells, which secrete the protective mucus layer, limiting the contact between bacteria and epithelial cells, and Paneth cells, which reside in the crypts of the small intestine and secrete bactericidal peptides as well as the predominant class of immunoglobulin IgA was also found in intestinal secretions (Cash et al., 2006). These mucosal immune responses are lessened when exposed to heat-treated bacteria in comparison to live organisms, suggesting that such mechanisms involve the metabolic products of bacterial activity as well as bacterial cell-receptor mediated sensing (Macpherson and Uhr, 2004). It should be noted that it is not only gram-negatives and lipopolysaccharides (LPS) that can induce inflammation; other cell components and metabolites can be involved, and there are also several gram-positive pathogenic (such as *Enterococcus,* which is often found as a contaminant in foods) and opportunistic pathogenic bacteria (such as *Bifidobacterium*) that can induce inflammation (Gonzalez-Navajas et al., 2008). An endeavour searching for the connection between gut microbiota and systemic inflammation showed that approximately 9% of the total variability of the microbiota was correlated to the pro-inflammatory cytokines IL-8 and IL-6 (Biagi et al., 2010). All taxa that showed a slightly positive association with either IL-6 or IL-8 belonged to the phylum *Proteobacteria* (Biagi et al., 2010). It is possible that low-grade systemic inflammation constitutes a common denominator in neurodegenerative and vascular diseases, possibly via detrimental effects on the vasculature and leading to a dysfunctional brain-blood barrier and inflammatory stimuli of the brain. Elevated peripheral inflammation could also affect brain inflammation by the “priming” of neurones, i.e. making them more prone to a pro-inflammatory response in the presence of tissue damage (Holmes et al., 2009). In addition, chronic inflammation during foetal and childhood development could negatively affect brain development and lower the “cognitive reserve” (Borenstein et al., 2006).

**Figure 1:**
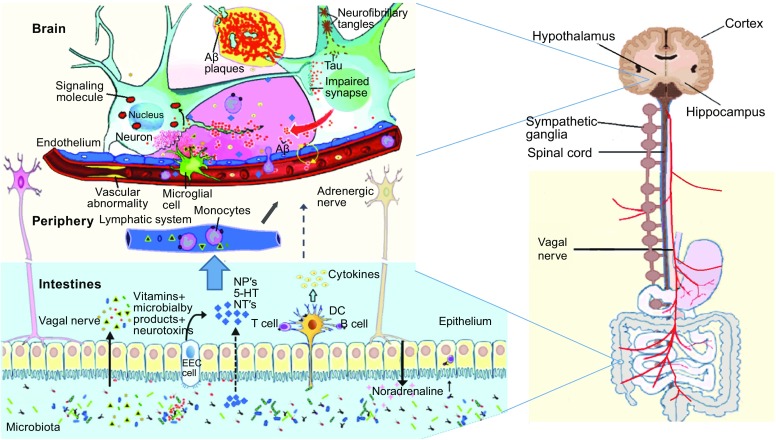
Schematic of some key players in the pathogenesis of AD. The gut microbiota regulation of neuro-inflammation and the hypothalamic–pituitary–adrenal (HPA) axis activity and may lead to AD. The bacterial products that gain access to the brain through the bloodstream and the area postrema, via cytokine release from mucosal immune cells, through the release of gut hormones such as 5-HT from EEC cells, or via afferent neural pathways, including the vagal nerve. NP: Neuropeptide; NT: Neurotransmitter; 5-HT: 5-hydroxytryptamine; DC: Dendritic cell; EEC: Enteroendocrine cell; Aβ: amyloid beta protein; AD: Alzheimer’s disease

The researchers noted that the affected AD brains are largely inflamed, glial cells rushing to the brain regions that become ill and trying to clean up waste products from cells and plaque, but once up to this area, they in fact are urged to liberate more Aβ harmful, precipitate in the formation of plaque, which attracts more accurate glial cells, and so on. According to Balin *et al* and other studies, amyloid proteins play a part in the disease, but only in response to the initial inflammation caused by the microbial infection, that is attacking the brain (Balin and Hudson, 2014). Recent study showed that Aβ may play a protective role in innate immunity and infectious or sterile inflammatory stimuli may drive amyloidosis and that Aβ oligomerization, a behavior traditionally viewed as intrinsically pathological, may be necessary for the antimicrobial activities of the peptide, which mean that Aβ serves to protect the brain from invading microbes (Kumar et al., 2016). In vitro study of *Chlamydia pneumoniae* showed that the infection of monocytes could stimulate innate and adaptive immune responses relevant to those in AD (Balin and Hudson, 2014; Lim et al., 2014; Little et al., 2014). Hoban *et al,* elucidated the mechanisms of the microbial influence by investigating changes in the homeostatic regulation of neuronal transcription of germ-free mice within the prefrontal cortex, and showed that the microbiome is necessary for appropriate and dynamic regulation of myelin-related genes (the formation of fatty sheathing that insulates nerve fibres), with clear implications for cortical myelination at an ultrastructural level (Hoban et al., 2016). Experiments by Lee *et al* also showed that the germ-free mice were protected from causing the case experimentally, similar to multiple sclerosis, characterized by the demise of myelin, which encases nerve fibers (Lee et al., 2011). There is also the possibility that these hypothetical pathways are tangled and that e.g. Aβ deposits in the cerebrovascular wall will elicit a peripheral inflammatory response that will in turn enhance brain inflammation (Fig. 1).

## Putative role of microbes’ products

The dysfunction of the gut epithelial barrier resulting from dysregulation of the brain–gut -microbiota axis could promote invasion of neuroactive substances, including neurotropic viruses, unconventional pathogens with prion–like properties, or slow neurotoxins (Hawkes et al., 2007). Some microbes can remain latent in the body with the potential for reactivation, the effects of which might happen years after early infection; and that people can be infected but not necessarily affected, such that ‘controls’, even if infected, but with symptoms (Itzhaki, 2014). It is remarkable that human microorganisms that produce amyloids such as CsgA, Aβ42, and other peptides that accrue in AD brains (Hill and Lukiw, 2015). For example, amyloids are related to fungal surface-structures and the new statement of amyloidogenic fungal proteins and diffuse mycoses in the blood of AD patients suggest that chronic fungal infection associates with high risk of AD (Hill et al., 2014). Moreover, *Escherichia coli* produce extracellular amyloids known as curli fibers, composed of the major structural sub-unit CsgA, are a common secretory component that facilitate surface adhesion, biofilm development and protection against host defences (Schwartz and Boles, 2013). A number of bacterial amyloid systems include *Pseudomonas, Streptomyces, Staphylococcus, Bacillus,* and others, propose that functional amyloids are an extensive phenomenon used by a wide diversity of microbiomes (Schwartz and Boles, 2013; Hill et al., 2014). Furthermore, bacterial enzymes may also produce neurotoxic metabolites such as D-lactic acid and ammonia (Galland, 2014). The direct neural communication between the gut and the brain occurs via the vagal nerve, as bacteria can stimulate afferent neurons of the enteric nervous system (ENS) (Forsythe et al., 2014). Vagal signals from the gut could also induce an anti-inflammatory reaction to protect the body from the infections caused by pathogens in a nicotinic acetylcholine receptor α7 sub-unit dependent manner (Grenham et al., 2011; Borre et al., 2014a; Forsythe et al., 2014; Mulak and Bonaz, 2015). Additionally, other human gut-resident *Cyanobacteria*-generated neurotoxins including β-*N*-methylamino-l-alanine (BMAA), saxitoxin, and anatoxin-α may further contribute to neurological diseases as amyotrophic-lateral sclerosis (ALS), the Parkinson-dementia complex of Guam, and AD especially over the course of aging (Brenner, 2013; Lakhan et al., 2013; Tran and Greenwood-Van Meerveld, 2013). Parodi *et al* demonstrated that rosacea patients have a significantly higher small intestinal bacterial overgrowth (SIBO) prevalence than controls, The effectiveness of SIBO eradication in rosacea may recommend that these bacteria play a role in the pathogenesis of rosacea lesions(Parodi et al., 2008). Moreover, patients who have rosacea are at increased risk of developing dementia, particularly AD (Nursing-Standard, 2016).

## Gut microbiota and metabolic diseases

High-fat diets and sedentary lifestyles have become major concerns throughout the world. The morbid conditions related to obesity such as abdominal obesity, insulin resistance and glucose intolerance, hypertension and dyslipidemia are together called the metabolic syndrome. Obesity and its co‐associated morbidities, namely cardiovascular disease (CVD), type‐2 diabetes mellitus (T2DM), fatty liver disease and hypertension are a great economic burden in affected countries. Of note, accumulating evidence suggests a mechanistic relation between the cholesterol metabolism in the brain and the formation of Aβ plaques in AD development (Martins et al., 2006). High blood sugar and body fat can place you at a higher risk for AD and blood restriction. Metabolomics study by Dumas *et al* suggested that gut microbiota may also play an active role in the development of complex metabolic abnormalities, such as susceptibility to insulin resistance and fatty liver disease (Dumas et al., 2006). Subsequent analysis of germ-free versus conventional mice on high-fat diet revealed that both insulin sensitivity and cholesterol metabolism are metabolic targets influenced by the gut microbiota (Rabot et al., 2010). Complementary clinical studies further demonstrated that raised circulating levels of the gut microbiota metabolite within subjects predicted increased cardiovascular danger independent of traditional cardiovascular risk factors (Wang et al., 2011). Increasingly, the role of CVD is also being recognized as an important etiologic hallmark of AD. Indeed, many studies were summarized findings on CVD and risk factors in the aetiology of AD and showed the importance of vascular pathology in AD (Jagust, 2001; de la Torre, 2004; de Bruijn and Ikram, 2014). Consequently, there is an increasing number of proofs suggesting that gut microbiota, through a variety of processes, can influence physiological processes important for the development of CVD (Hazen and Smith, 2012).

## Obesity increases the risk of cognitive impairment or mental decline

**Figure 2:**
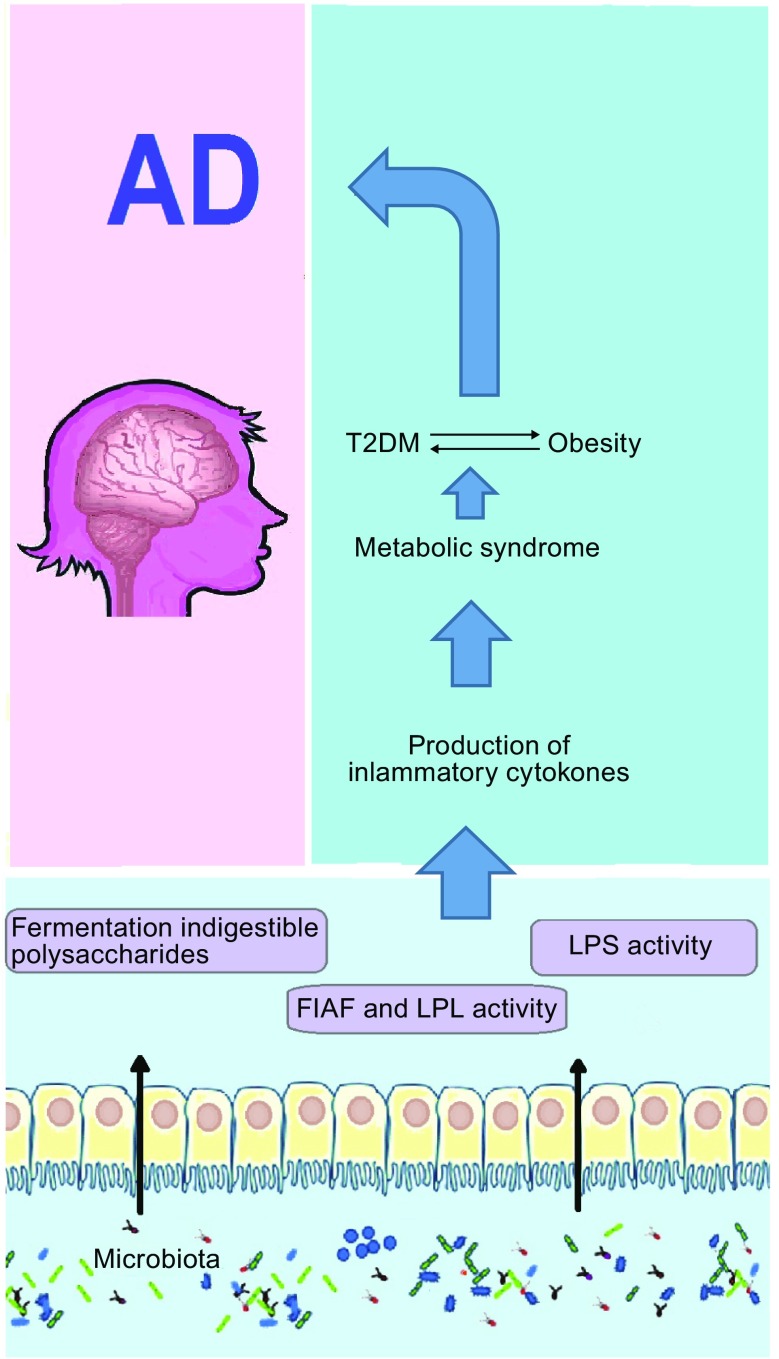
The links between gut microbiota and metabolic diseases, as obesity and further development of T2DM with AD. FIAF: fasting-induced adipocyte factor; LPS: lipopolysaccharide; LPL: lipoprotein lipase; T2DM: type 2 diabetes mellitus; AD: Alzheimer’s disease

It is generally believed that gut microbiota control obesity, the major cause of T2DM, which has recently been linked with AD development (Pasinetti et al., 2011). Recent studies have also found the role of gut microbiota in the control of brain function directly by tryptophan metabolism, production of microbial metabolites, microbial neurotransmitters and bacterial cell wall sugars and bile acids (Swann et al., 2011; Collins et al., 2012; Tremaroli and Backhed, 2012). Tremaroli *et al* found conclusive evidence that gut microbiota could influence the activity of lipoprotein lipase (LPL) -the key enzyme involved in the release of fatty acids from triglyceride-rich lipoproteins in muscle, heart, and fat- by affecting the expression of fasting-induced adipocyte factor protein (FIAF)—key inhibitor of LPL activity and plays an important role in preventing obesity- that was over expressed in the germfree mice and reduce storage of triglycerides in the adipose tissue (Tremaroli and Backhed, 2012). Upregulation of adipocyte LPL activity leads to increase cellular uptake of fatty acids and adipocyte triglyceride accumulation. Consequently, suppression of intestinal FIAF by microbes promotes adiposity through upregulation of LPL activity in adipocytes and increased hepatic lipogenesis were found to enhance the accumulation of calories harvested from the diet into fat then storage in the liver, which is the main cause of insulin resistance in obesity (Fig. 2). Moreover, specific bacterial taxa of the gut microbiota are involved in nutrient uptake and energy homeostasis and may lead to low grade inflammation induced by LPS, causing activation of the innate immune response. This low grade inflammation is connected to low, but constant levels of LPS in the circulation and to increased levels of adiposity and insulin resistance (obesity and T2DM) (DiBaise et al., 2008). Additionally, previous literature demonstrated that ileal inflammation, decreased LPS activity, and increased innate immune system activation was observed in rats susceptible to weight gain as compared to the obesity resistant rats (de La Serre et al., 2010).

## Type-3 diabetes

New evidence has come to light concerning the different expressions of diabetes, as well as its connection to other diseases. It used to be supposed that there were two types of diabetes: type-1 and type-2. However, the idea that AD might be type-3 diabetes was proposed ten years ago (Martins et al., 2006). Some studies suggested that AD progresses because of the brain developing resistance to insulin, which in turn prevents proper lipid uptake. Over time, these lipids build up in the brain rather than properly absorb, which results in increased stress and inflammation, as well as the symptoms usually related with dementia (de la Monte, 2014; Rani et al., 2016). Given the evidence provided by animal models of the strong relationship between T2DM and AD. When the authors blocked the path of insulin to rats’ brains, their neurons deteriorated, they became physically confused and their brains showed all the signs of AD. Furthermore, the study provided proof that T2DM and AD aggravate each other and suggested several potential mechanisms underlying the two disorders, such as, Aβ clearance by insulin degradation enzyme (IDE), glucose metabolism, O-GlcNAcylation, Aβ aggregation by advanced glycation end products (AGEs), oxidative stress, circulating cortisol, and cerebral vascular insufficiency, as well as inflammation, and aging. In fact, people with diabetes have double the risk of developing AD. This does not mean that everyone who has diabetes will eventually develop AD, or that all people with AD have diabetes. It is important to report that there is an important link between diabetes and AD, and it is therefore common that pathophysiology probably constitutes a major underpinning of late-onset sporadic AD, and a novel therapeutic approach targeting this pathological process could contribute to the development of a more efficient and effective treatment for AD (Park, 2011; de la Monte, 2014).

## Aging

Alzheimer’s cases show a dramatic increase with age, affecting about 1% of the population aged between 65-69 years up to 30-40% in the oldest individuals (Gatz et al., 2006). Overall occurrence in the population above 65 years of age is estimated to 6-8% and is expected to increase significantly worldwide due to changing demographic profiles with an ever-increasing proportion of elderly (Ferri et al., 2005). Indeed, as adulthood approaches, the gut microbiota stabilizes, becomes more diverse, and can resist detrimental environmental elements such as antibiotics and stress by restoring its diverse and stable ‘normal’ core microbiota (Palmer et al., 2007; Rajilic-Stojanovic et al., 2011). However, as we age, the diversity and stability of our microbiota will decline (Biagi et al., 2010), moreover there is an associated increase in number, size and activation of microglia that can lead to an increased inflammatory tone named ‘inflammaging’ (Franceschi, 2007; von Bernhardi et al., 2015). There is high heterogeneity of microglia in several neurodegenerative diseases, including AD and those phenotypes share common characteristics with aging (Bachstetter et al., 2015). In normal aging, close crosstalk with astrocytes, neurons and other brain cells serve crucial functions as the scavenger system of the CNS, providing beneficial functions such as tissue repair in the CNS. However, chronic, dysregulated activation of microglia can lead to elevated inflammatory tone ultimately resulting in the malfunction or damage of brain cells. The role of the microbiota in addition to what drives this dysregulation has yet to be fully understood.

## AD mice and gut microbiota

A recent experimental study conducted by Harach *et al* observed marked differences of the gut microbiota composition between aged AD mice and healthy WT mice and strongly advocated that a distinct microbial constitution in AD mice may play a role in the development of cerebral Aβ amyloidosis (Harach et al., 2015). In this study, the authors demonstrated that the abundance of the two major phyla (Firmicutes andBacteroidetes) in the fecal microbiota is dramatically altered by host genetics. At genus level, *Allobaculum* and *Akkermansia* were decreased in transgenic CONVR-APPPS1 mice and unclassified genera of *Rikenellaceae* and *S24-7* were increased. Reduced levels of *Akkermansia* have been associated with obesity and type 2 diabetes in mice (Everard et al., 2013), and the prebiotic-induced restoration of *Akkermansia* in the gut resulted in a lower fat-mass gain and decreased systemic inflammation (two discussed risk factors in the development of AD pathology). Furthermore, the relative abundance of several bacterial genera was correlated with the amount of soluble Aß42 in the brain (Harach et al., 2015).

## Delay the process of neurodegeneration

### Probiotics and prebiotics

The increased understanding of the impact of the gut microbiota on human health resulted in attempts to manipulate its composition by the use of probiotics and prebiotics, from both prophylactic and therapeutic perspectives. Probiotics are defined as live microbial organisms that, when consumed in adequate quantities, confer health benefits to the host (Berg, 1998). Prebiotics are chemical substances, usually oligosaccharides, acting as substrates specifically for the host’s autochthonous probiotic bacteria, and thus promoting their growth. Prebiotics are selected as being non-digestible by the host and non-metabolizable by non-probiotic gut bacteria, but stimulating for bifidobacteria and lactobacilli (Gibson et al., 2004; Hamilton-Miller, 2004). A food is considered synbiotic when it contains a prebiotic and a probiotic ingredient. The species *Lactobacillus, Bifidobacterium* and *Streptococcus* are the most commonly studied to date because they are common and are ‘natural’ members of the intestinal microbiome (Gordin and Gorbach, 1992; Berg, 1998). Moreover, these bacteria do not necessarily need to be alive, as products of the bacteria, such as cell walls and bacterial DNA, can modulate the profile of the gut microbiota and immune responses (Agostoni et al., 2004; Thibault et al., 2004). Their main beneficial effects are to function as the first barrier to pathogenic organisms by adherence, to produce substances that have antimicrobial effects, and to stimulate the immune processes in the host (Floch, 2005; Chermesh and Eliakim, 2006). Furthermore, certain strains of lactic acid bacteria (LAB) produce the complex vitamin cobalamin (or vitamin B12) which directly associated with AD, based on many of the studies which showed that vitamin B12 levels are lower in AD individuals compared with healthy subjects (LeBlanc et al., 2013; Chen et al., 2015). Additionally, fermented milks with high levels of B-group vitamins (such as folate and riboflavin) can be produced by LAB-promoted and possibly *Bifidobacteria*-promoted biosynthesis (LeBlanc et al., 2013). As the pathogenesis of different diseases is diverse, the mechanisms by which bacteria effect disease processes are unique. All the same, probiotic development shows great capacity for rebuilding microbiotas and restoring health, especially for some individuals. Therefore, the practical application portability of species beneficial in one illness will not necessarily hold for another. As for the prebiotics, inuline supplementation was reported to be able to increase the viable count of bifidobacteria in constipated elderly, while the frequency of the detection of enterobacteria decreased with the treatment. The ingestion of inuline improved constipation in 9 out of 10 subjects (Kleessen et al., 1997). It was reported that fructo-oligosaccharide ingestion (Guigoz et al., 2002; Bouhnik et al., 2007), as well as the supplementation of a galacto-oligosaccharide mixtures are able to significantly increase the numbers of bifidobacteria at the expense of less beneficial groups (Vulevic et al., 2008).

### NSAIDs

Several epidemiological studies suggested that NSAIDs may delay the onset of AD for up to five or more years, and are even able to prevent the onset of AD in patients with mild cognitive impairment (MCI) or in healthy elderly subjects at risk of developing AD (Imbimbo et al., 2010). Thus, it could be hypothesized that the chronic use of NSAIDs may be beneficial only in the very early stages of the AD process in coincidence of initial Aβ deposition, microglia activation and consequent release of pro-inflammatory mediators. When the Aβ deposition process is already started, NSAIDs are no longer effective and may even be detrimental because of their inhibitory activity on chronically activated microglia that on long-term may mediate Aβ clearance (Imbimbo et al., 2010). In conclusion, these studies indicate that there is a dose-response relationship between NSAID use and the relative risk of AD, with longer periods of use related to reduce relative risks of AD. Based on these studies, the relative risk of AD appears to be 25-50% lower in groups of individuals with long-term (2 years or more) NSAID use (in t’ Veld et al., 2001). The reduction in risk also appears to be restricted to AD; no protective effect against vascular dementia was noted. The hypothesis is that the inflammatory response to the accumulating Aβ and tau deposits worsens the pathological process and that NSAIDs may alleviate the process by inhibiting the inflammatory response and/or inhibiting glutamate toxicity (Casper et al., 2000; Imbimbo et al., 2010).

### Grape seed

Grape seed polyphenol extract (GSPE) was widely considered a dietary supplement with widespread health benefits. Many studies recently demonstrated the potential efficacy of GSPE in protecting against neuropathology and cognitive impairment in animal models of AD and tau-mediated neurodegenerative disorder (Wang et al., 2008; Santa-Maria et al., 2012). The growing body of experimental, preclinical, and clinical evidence supporting GSPE exerting beneficial biological activities in multiple medical conditions, has led to increased interest in its bioavailability, metabolism, and distribution of the primary GSPE phenolic constituents, including gallic acid, epicatechin, proanthocyanidin dimers, and larger oligomers. Interestingly, evidence suggests that intestinal microbiota significantly contribute to GSPE metabolism/absorption, as catechin and epicatechin, major components of GSPE, are both metabolized by colonic microbiota fermentation (Aura et al., 2002; Cueva et al., 2013). Wang *et al* demonstrated that intestinal microbiota may contribute to the protective activities of GSPE in neurodegenerative disorders, and in other diseases, by converting proanthocyanidin components from GSPE to phenolic acid metabolites capable of accumulating in target tissues, such as the brain, and of exerting disease-modifying activates (Wang et al., 2015). Table 2 summarize the methods described above.

**Table 2:** Some methods that using to delay the process of neurodegeneration

**Products**	**Description**	**Components**	**Foods contain them**
Probiotic	Live microorganisms confer a health benefit and boost the host immunity	*·* *Lactobacillus acidophilus* *·* *Lactobacillus casei* *·* *Lactobacillus reuteri* *·* *Lactobacillus plantarum* *·* *Lactobacillus rhamnosus* *·* *Bifidobacterium animalis* *·* *Bifidobacterium infantis* *·* *Bifidobacterium lactis* *·* *Bifidobacterium longum*	Yogurt, Soy yogurt fermented dairy products Kombucha^a^, Kimchi^b^ Miso^c^, Sauerkraut^d^
Prebiotic	Chemical substances, nondigestible foods that make their way through our digestive system and help good bacteria grow and flourish. Prebiotics help feed and keep beneficial bacteria healthy	Mostly come from carbohydrate fibers called oligosaccharides	Bananas, Onions, Garlic, Leeks, Asparagus, Whole wheat, Barley, Rye, Inulin^e^
NSAIDs	A drug class that groups together drugs: provide analgesic (pain-killing) and antipyretic (fever-reducing) effects, and, in higher doses, anti-inflammatory effects	Aspirin, indomethacin, ibuprofen, ketoprofen, diclofenac, piroxicam, celecoxib, nimesulid	Apples, Avocados, Blueberries, Broccoli, Cauliflower, Cherries, Chili peppers, Cucumbers, Dates, Eggplant, Figs…
GSPE^f^	An industrial derivative of whole grape seeds used as a dietary supplement with widespread health benefits	Catechin, gallic acid, epicatechin, proanthocyanidin dimers, larger oligomers	Grape seeds

^a^Kombucha—slightly effervescent drink that is brewed with tea and sugar and fermented into a liquid. This beverage originated in China nearly 2000 years ago

^b^Kimchi—a traditional Korean lacto—fermented condiment made from cabbage

^c^ Miso is made by adding an enzymatic culture to a soybean base and often a grain

^d^Sauerkrautis cabbage that has been salted and lacto-fermented over a period of weeks

^e^ Inulin is a natural prebiotic fiber that is found in over 36,000 plants worldwide

^f^GSPE—grape seed polyphenol extract

### Critical challenges

During the last three decades, Alzheimer’s research has not only made remarkable progress in understanding the disease but also has recruited some of the best scientists in the world. The prospect of delaying or preventing the onset of symptoms is feasible and within our grasp. However, this mission must surmount a number of barriers, which contain inadequate funding of research, high cost of clinical studies, lack of suitable infrastructure, better models, antiquated administrative structure of discovery programs, and arcane decision-making systems for selecting and funding innovative ideas. Recently, the only way to know for certain that someone has AD is to examine an autopsy of their brains tissue after death. The crucial challenge for the AD researches is how to understand the process of neurodegenerative disorders throughout the patient’s lifetime. Additionally, the lack of efficient cultivation techniques stems from many factors that largely remain unknown (Lopez et al., 2015). Hence, molecular ecology and metagenomics have significantly increased our knowledge of the genetic diversity and have led to interesting hypotheses (Hugenholtz and Tyson, 2008). The advanced techniques have also revealed how far we are from measuring the full extent of genetic diversity encoded by microbial life (Hugenholtz and Tyson, 2008; Pignatelli et al., 2008).

## Summary

Microbial colonization of the gut plays a key role in the postnatal development and maturation of the immune, endocrine and even neural systems, these processes are key factors underpinning CNS signalling. Indeed, understanding the gut microbiota is important in relation to inflammation and metabolic diseases that have a direct relation to the AD pathogenesis. Moreover, comparative analysis of gut microbiota may enable further novel vision into the complex biology of AD, which is very important in order to take preventive measure such as early diagnosis, identification of new therapeutic targets and development of novel drugs. Thus, modulation of gut microbiota (by probiotics or other dietary intervention) or direct targeting of gut microbiota enzymes (by pharmacological inhibitors or activators) may be a growing area for pharmaceutical and functional food industries, with the goal of decreasing the widespread growth of adiposity, insulin resistance, AD, and other metabolic diseases.
